# The Identification and Expression Analysis of the *Liriodendron chinense F-Box* Gene Family

**DOI:** 10.3390/plants13020171

**Published:** 2024-01-08

**Authors:** Shichan He, Lin Xu, Weihuang Wu, Jiaji Zhang, Zhaodong Hao, Lu Lu, Jisen Shi, Jinhui Chen

**Affiliations:** 1 State Key Laboratory of Tree Genetics and Breeding, Co-Innovation Center for Sustainable Forestry in Southern China, Nanjing Forestry University, Nanjing 210037, China; 2 Key Laboratory of Forest Genetics and Biotechnology of Ministry of Education, Nanjing Forestry University, Nanjing 210037, China; 3 College of Landscape Architecture, Nanjing Forestry University, Nanjing 210037, China

**Keywords:** *L. chinense*, *F-box* gene family, expression profiles, somatic embryogenesis, drought stress

## Abstract

The *F-box* gene family is one of the largest gene families in plants, and it plays a crucial role in regulating plant development, reproduction, cellular protein degradation, and response to biotic and abiotic stresses. Despite their significance, a comprehensive analysis of the *F-box* gene family in *Liriodendron chinense* and other magnoliaceae species has not been reported. In this study, we report for the first time the identification of 144 full-length *F-box* genes in *L. chinense*. Based on specific domains and phylogenetic analyses, these genes were divided into 10 distinct subfamilies. We further analyzed their gene structure, conserved domain and chromosome distribution, genome-wide replication events, and collinearity. Additionally, based on GO analysis, we found that *F-box* genes exhibit functional specificity, with a significant proportion of them being involved in protein binding (GO:0005515), suggesting that *F-box* genes may play an important role in gene regulation in *L. chinense*. Transcriptome data and q-PCR results also showed that *F-box* genes are involved in the development of multiple tissues in *L. chinense*, regulate the somatic embryogenesis of *Liriodendron* hybrids, and play a pivotal role in abiotic stress. Altogether, these findings provide a foundation for understanding the biological function of *F-box* genes in *L. chinense* and other plant species.

## 1. Introduction

Ubiquitin is a small protein molecule that is found widely within eukaryotic cells. Its fundamental role is to promote protein degradation and DNA damage repair, and it achieves this by catalyzing a series of enzymatic reactions to attach to target proteins. The ubiquitin-mediated protein degradation pathway involves three enzymes: ubiquitin-activating enzyme E1, ubiquitin-conjugating enzyme E2, and ubiquitin ligase E3. Among these enzymes, E3 is the most intricate and varied in structure and number [1]. One of the most extensively studied E3 ubiquitin ligases is the SCF complex, which is comprised of Rbx1, Skp1, Cullin, and F-BOX. Rbx1, Skp1, and Cullin form the framework of the complex, while different F-BOX are capable of recognizing specific substrates, which are subsequently degraded via targeted degradation [2,3].

The F-BOX, which contains a conserved F-BOX motif of approximately 40 amino acids, is a member of one of the largest protein families present in eukaryotes [4]. Since the discovery of the first F-BOX (Cyclin F) in humans [5], numerous F-BOX have been identified based on the conserved motif of its N-terminal [6]. The number of *F-box* genes differs across species, and plants typically have a greater number of *F-box* genes than animals. For instance, while *Caenorhabditis elegans* has the highest number of *F-box* genes in animals with a count of 326, there are only 69 and 22 *F-box* genes in humans and *Drosophila*, respectively [6,7]. On the other hand, plants have a greater number of *F-box* genes, such as 694 in *Arabidopsis thaliana* [8], 972 in *Medicago truncatula* [4], 592 in *Gossypium hirsutum* [9], 480 in soybean (*Glycine max*) [10], 320 in poplar (*Populus trichocarpa*) [8], 285 in chickpea (*Cice rarietinum*) [11], 226 in pear (*Pyrus bretschneideri*) [12], 126 in barley (*Hordeum vulgare*) [13], and so on. While the *F-box* gene family consists of numerous members, only a limited number of these genes have undergone functional characterization. For example, in *Arabidopsis*, there are 694 *F-box* genes, but just 41 (equivalent to 5.9% of the total) have to date undergone functional characterization [14]. This small number of *Arabidopsis* F-BOX with known functions suggests that more extensive research is needed to fully understand *Arabidopsis F-box* genes. In addition, many plant *F-box* genes, including those from *Arabidopsis*, are inactive and, under low functional constraints, likely result from a novel deleterious duplication mechanism of these genes [15,16].

Besides the N-terminal F-BOX domain, *F-box* genes generally also contain other conserved motifs at their C-terminal regions, such as Kelch repeats, Leucine-rich repeats (LRR), the F-BOX-associated domain, the Tubby domain, and others. The *F-box* gene family is divided into various subfamilies based on differences among these conserved motifs, including *FBL* (LRR), *FBA* (Associated domain), *FBK* (Kelch repeats), *FBO* (Other domain), *FBT* (Tubby), *FBP* (PP2 domain), *FBD* (FBD domain), and *FBX* (only F-BOX domain), amongst others [9,10,11,12,13].

As one of the largest gene families in plants, the *F-box* gene family plays a crucial role in regulating plant growth and development [12,17], as well as abiotic stresses [4,9,12] and signal transduction of plant hormones [18,19]. For example, miR394 regulates the F-BOX LCR (LEAF CURLING RESPONSIVENESS), which in turn modulates stem cell regulation. Overexpression of *LCR* can cause the termination of *A. thaliana* shoot tip meristem development, while *LCR* mutations can rescue the stem cell deletion phenotype of miR394 mutants, leading to the formation of a normal shoot tip meristem [20]. Furthermore, the *F-box* gene *UCL1* (*UPWARD Curly Leaf 1*) is responsible for regulating the leaf curl in *A. thaliana* [21], and *LC4* (*LEAF INCLINATION 4*) influences leaf tilt angle in rice, impacting the overall morphogenesis of the plant [22]. Apart from this, *F-box* genes have been known to be involved in the plant’s response to abiotic stresses, and there is extensive literature available on this subject. For example, arsenic (As) is a non-biodegradable inorganic contaminant that is highly toxic to both plants and animals. One such *F-box* gene associated with As-stress, ASRF, has been found to have a significant association with As-sensitive mutants. The seedlings of *asrf* mutants show increased sensitivity to arsenate (ASV) stress, highlighting the indispensable role of *F-box* genes in plants’ responses to stress [23]. In wheat, the *F-box* gene *TAFBA1* was shown to enhance the resistance of transgenic plants to heat stress by interacting with other proteins, such as TaASRP1, to improve the antioxidant levels of the enzyme and regulate gene expression [24]. *TAFBA1* has also been found to be associated with drought stress [25]. Additionally, in grapes, the *F-box* gene BIG-24.1 is strongly induced by different abiotic stresses, including UV-C exposure, injury, and treatment with salicylic acid, methyl jasmonate, ethylene, and abscisic acid. BIG-24.1 was also found to be stimulated by non-host bacteria and endophytic rhizobium rhamnolipid in berry and grape cells infected with Botrytis cereus. Furthermore, analysis of the BIG-24.1 promoter sequence revealed the existence of several regulatory elements involved in the activation of plant defense responses [26]. These results indicate that the *F-box* gene plays different roles in plant growth and development and in regulating plant responses to environmental stress. From regulating stem cell development to influencing morphogenesis and mediating plant stress response, the *F-box* gene provides a promising target for cultivating stress-tolerant plants under changing climate conditions.

*Liriodendron* is a basal genus of angiosperms within the Magnoliaceae family, consisting of two species: *L. tulipifera*, which is primarily distributed in North America, and *L. chinense*, mainly found in East Asia. Additionally, *L. sino-americanum* is a hybrid species formed by crossing these two species [27]. *Liriodendron* is an extensively cultivated ornamental tree species due to its beautiful tree shape and exceptional ability to withstand environmental adversity [28]. The 2019 *L. chinense* genome [29] has provided valuable resources for genome-wide analyses of gene families that shape the growth, development, and response to environmental stressors of this tree species. Several gene families have been studied, including *CBF* [30], *WRKY* [31], *MYB* [32], *TPS* [33], *PIN* [34], etc. Here, we conducted a genome-wide gene family analysis of *F-box* genes in *L. chinense*, exploring various aspects such as conserved domains, gene structure, phylogenetic tree, chromosome locations, gene replication, and collinearity. Additionally, we analyzed gene ontology and predicted the subcellular localization of the *F-box* gene family in *L. chinense*. Importantly, to further examine the function of the *F-box* gene family in *L. chinense*, we combined transcriptome and qRT-PCR to analyze the expression of *F-box* genes in different tissues during somatic embryonic development and under stress treatment. These findings contribute to our understanding of the mechanisms underlying plant growth, development, and response to environmental stress. Moreover, they provide potential avenues for the cultivation of desirable seedlings.

## 2. Results

### 2.1. Genome-Wide Identification, Characteristics, and Classification of F-Box Genes in L. chinense

**Figure 1:**
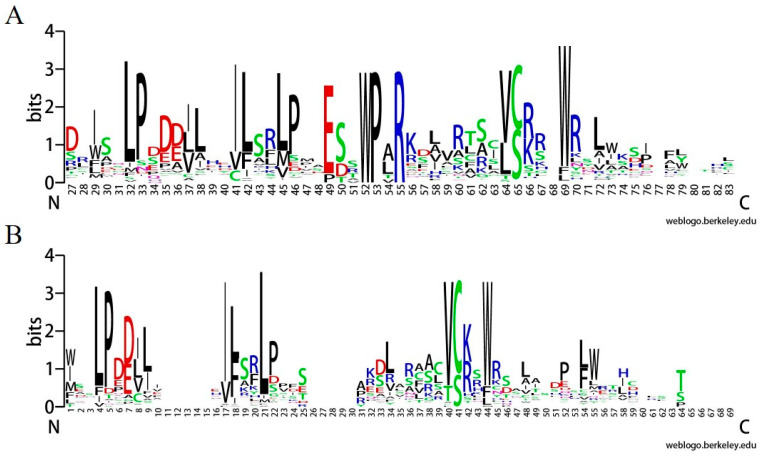
WebLogo based on the alignments of the F-BOX motifs and F-BOX-LIKE motifs from 144 F-BOX proteins in *L. chinense*. (**A**) represents F-BOX motifs, and (**B**) represents F-BOX-LIKE motifs.

Based on our analysis using HMMER (v2.41.2), we identified 163 *F-box* genes in *L. chinense*. We then screened these genes using the SMART (2020), Pfam, and CDD databases and identified 144 *F-box* genes with conserved domains. We used TBtools software (v2.034) to extract the F-BOX and F-BOX-LIKE conserved domain sequences of these 144 F-BOX in *L. chinense*, and the two conserved motifs were visualized using the Weblogo online software (v2.8.2). The F-BOX motif is typically located at the N-terminal of F-BOX and has a length of approximately 40 to 50 amino acids. The conservative F-BOX sequence of *L. chinense* F-BOX contains approximately 50 amino acid residues, including an extremely conservative tryptophan residue (W) at the 43rd position, as well as other conservative amino acid residues such as leucine (L) at the 6th position, proline (P) at the 7th and 27th positions, tryptophan (W) at the 26th position, arginine (R) at the 29th position, valine (V) at the 38th position, and more (Figure 1A). These conserved amino acid residues may interact with tryptophan residues to maintain the α helix structure of the F-BOX motif of *L. chinense* [5] and enable the proteins to perform their specific functions. We also analyzed the F-BOX-LIKE conserved motif, which is also located at the N-terminal of the F-BOX and contains approximately 60 amino acid residues. In addition to an extremely conserved tryptophan residue (W) at the 41st position, other conserved amino acid residues include leucine (L) at the 4th and 21st positions, proline (P) at the 5th position, isoleucine (I) at the 17th position, valine (V) at the 40th position, and more (Figure 1B). Our analysis shows that the conserved amino acid residues of these two conserved motifs are largely consistent.

We also analyzed some basic characteristics of the 144 *F-box* genes. We found that the shortest protein length was 95 amino acids with a molecular weight (MW) of 10.71 kDa, while the longest protein length was 1582 amino acids with a MW of 181.88 kDa. The differences were quite large, with an average length of 461 amino acids and an average MW of 51.97 kDa. Additionally, we observed that among the 144 *F-box* genes, the isoelectric points (pI) of the corresponding proteins of 65 genes were acidic, while the remaining 79 genes had alkaline protein products (Table S1).

**Figure 2:**
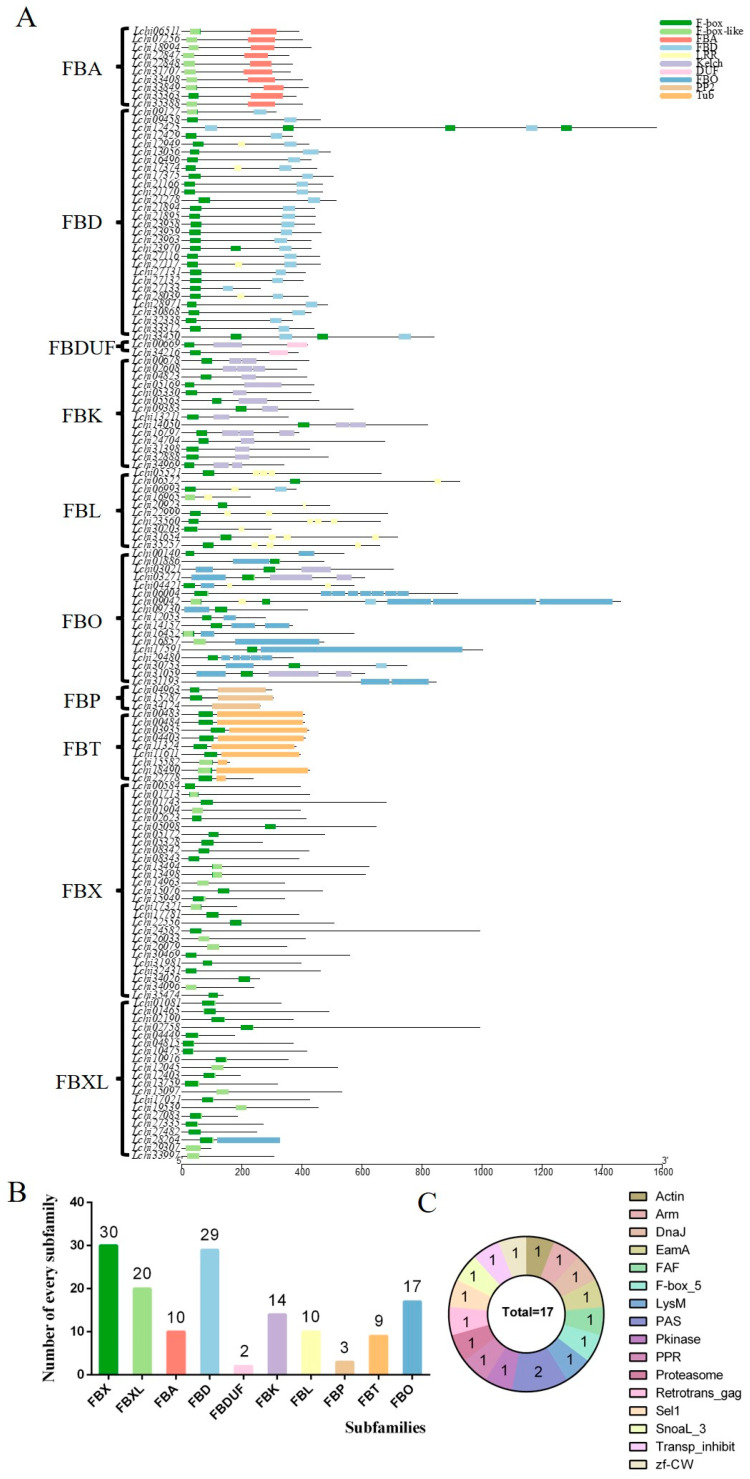
Conserved domain of the *L. chinense F-box* gene family. (**A**) represents the conserved domain of 144 *F-box* genes; (**B**) represents the number of members of different subfamilies; and (**C**) represents the conserved domain and the number of members contained in the *FBO* family, the number in the fan represents the number of members.

**Figure 3:**
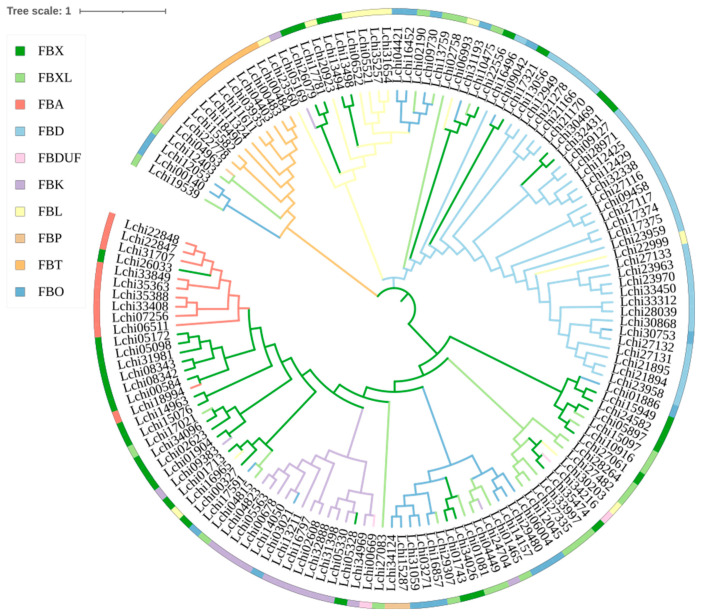
Phylogenetic tree of the *L. chinense F-box* family gene. The ClustalW program in MEGA-X software (v10.1.8) was used to perform multiple sequence alignments on the full-length amino acid sequences of 144 *F-box* genes in *L. chinense*. The phylogenetic tree was constructed using the maximum likelihood (ML) method. The Jones-Thornton-Taylor (JTT) model algorithm was used to estimate 1000 bootstrap repeats.

We classified the 144 *F-box* genes based on the C-terminal conserved domain types of each *F-box* gene, and they were divided into 10 subfamilies, including: *FBX* (only F-BOX), *FBXL* (F-BOX-LIKE), *FBA* (associated domain), *FBD* (FBD domain), *FBDUF* (domain of unknown functions), *FBK* (Kelch repeats), *FBL* (Leucine-rich repeats), *FBP* (PP2), *FBT* (Tubby), and *FBO* (other domain) (Figure 2A,B). The *FBO* subfamily members include Actin, Arm, DnaJ, EamA, LysM, Pkinase, Sel1, SnowaL_3, zf-cw, and others (Figure 2C). To verify our classification, we further constructed a phylogenetic tree using the full-length amino acid sequences of the *F-box* genes, which showed that members of the same subfamily tended to cluster on the same branch. For example, the *FBD*, *FBA*, *FBK*, and *FBT* genes were found to cluster closely together (Figure 3). This indicates that our classification based on the C-terminal conservative domain has some credibility.

**Figure 4:**
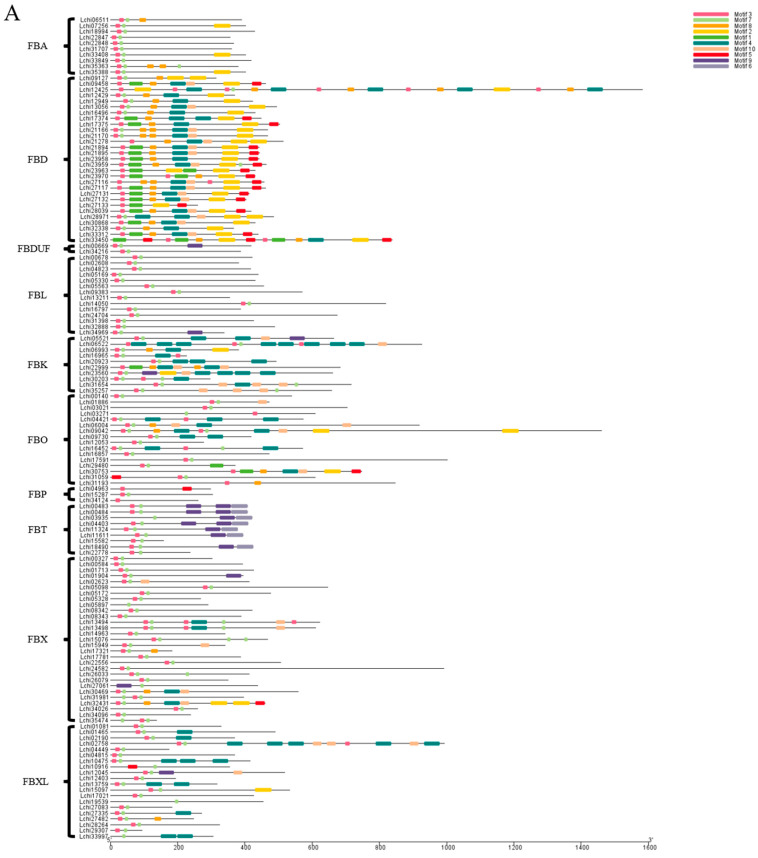

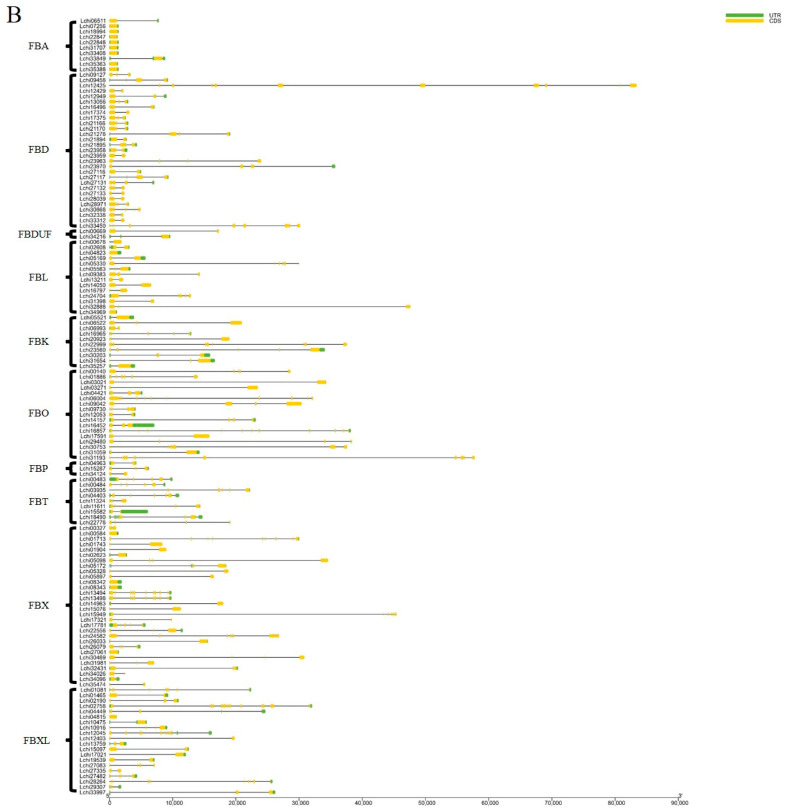
Gene structure and conserved motif analysis of the *L. chinense F-box* family. (**A**) represents the motifs of *F-box* genes in different *F-box* subfamilies, in which different color frames represent different motifs; (**B**) represents the gene structure of the *F-box*, where the green box represents the UTR, the yellow box represents the CDS, and black lines represent introns.

We also analyzed the gene structure and motif of the 144 *F-box* genes. We found that the number of motifs tended to be consistent within the same gene family. For instance, the *FBD* and *FBL* subfamilies had a relatively large number of motifs, while the *FBA* and *FBK* subfamilies had a relatively small number of motifs. In contrast, the number of introns on the 144 *F-box* genes was highly variable, ranging from 0 to 16, while the number of exons ranged from 1 to 17. The numbers of UTRs ranged from 0 to 2 (Figure 4, Table S2).

### 2.2. Chromosomal Locations, Gene Duplication Events, and Synteny Analysis in the L. chinense F-Box Gene Family

**Figure 5:**
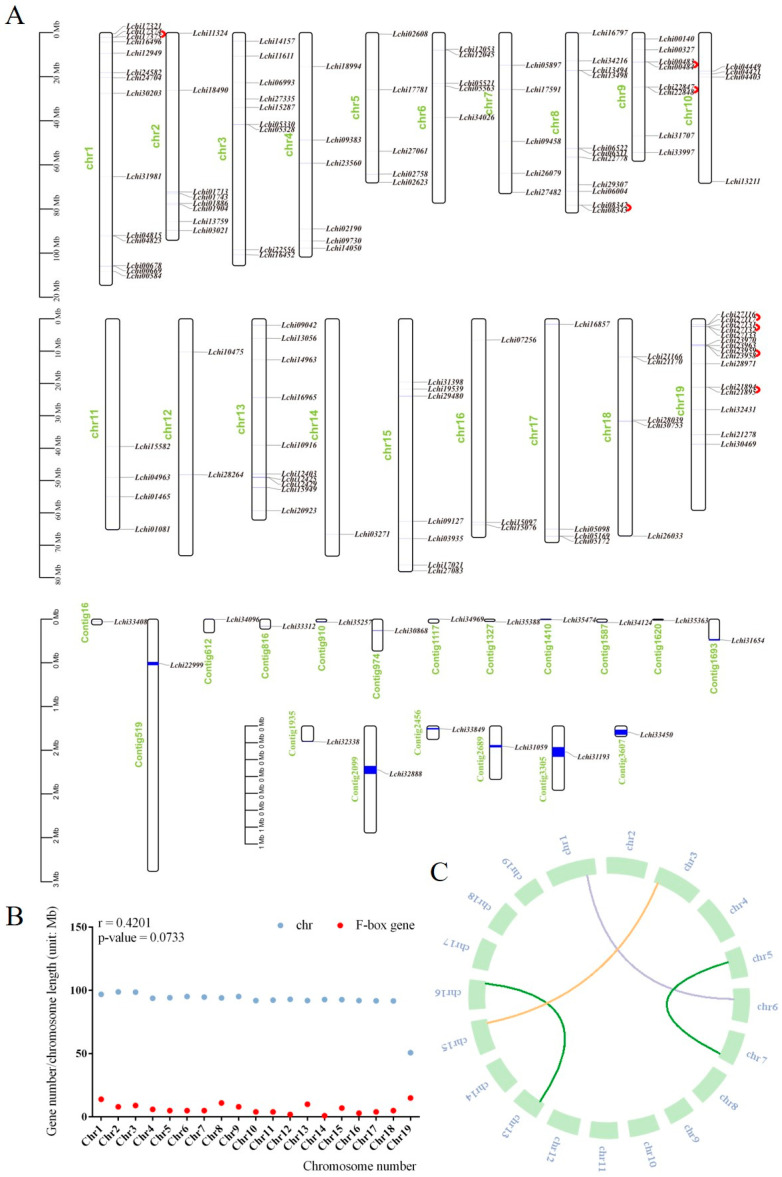
Chromosome distribution, gene tandem duplication, and collinearity analysis of the *F-box* gene in *L. chinense*. (**A**) represents the distribution of *F-box* genes on chromosomes, and the red arc represents tandem repeat genes. The blue line represents the gene, and its thickness represents the length of the gene on the chromosome. (**B**) represents the correlation analysis between *F-box* gene number and chromosome length; (**C**) represents the collinearity of the *F-box* gene within *L. chinense*, Lines of different colors represent different subfamilies.

Based on the genomic annotation document, we found that *F-box* genes were distributed on all chromosomes of *L. chinense*. Among them, 18 *F-box* genes were distributed on several different contigs, while the rest were distributed on different chromosomes of *L. chinense*. Chromosome 1 had the most *F-box* genes, with a total of 14, followed by Chromosome 8. In contrast, Chromosome 14 had the least number of *F-box* genes, with only one (Figure 5A). Correlation analysis of 126 *F-box* genes that were distributed on chromosomes and chromosome length revealed that chromosome length showed no significant correlation with the distribution number of *F-box* genes (Figure 5B). We also found that among these 126 *F-box* genes, there were eight tandem repeating pairs. These pairs included *Lchi00483/Lchi00484*, *Lchi08342/Lchi08343*, *Lchi17374/Lchi17375*, *Lchi21894/Lchi21895*, *Lchi22847/Lchi22848*, *Lchi23958/Lchi23959*, *Lchi27116/Lchi27117*, and *Lchi27131/Lchi27132* (Figure 5A).

We also analyzed the collinearity of these 144 *F-box* genes and found a total of four pairs of linear relationships. Two of these pairs belonged to the *FBX* subfamily, one belonged to the *FBT* subfamily, and one belonged to the *FBK* subfamily (Figure 5C).

### 2.3. Subcellular Localization, GO, KEGG Enrichment Analysis, and Protein Interaction Prediction of the F-Box Gene in L. chinense

**Figure 6:**
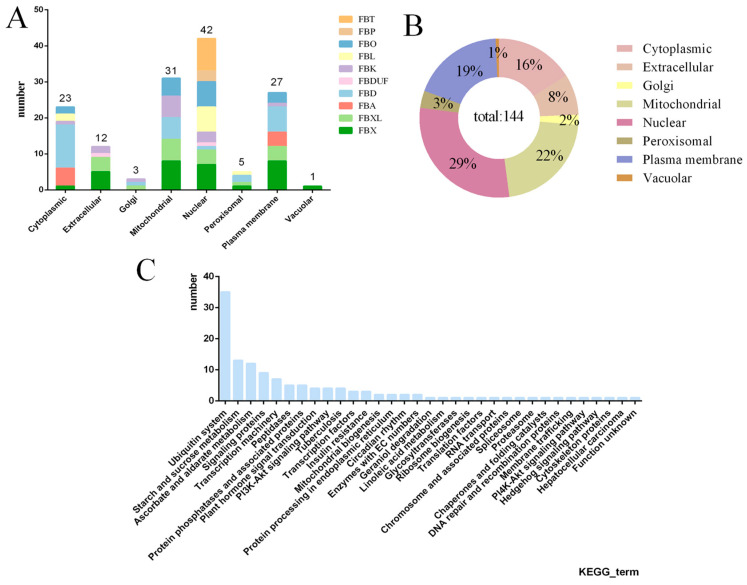
Subcellular prediction and KEGG enrichment analysis of *L. chinense F-box* family genes. (**A**) represents the prediction of the subcellular structure of *F-box* genes in different subfamilies, and the number on the column represents the number of *F-box* genes. (**B**) represents the proportion of *F-box* genes predicted by different cell structures. (**C**) represents the KEGG enrichment pathway analysis of *F-box* family genes.

To analyze the potential functions of these 144 *F-box* genes in *L. chinense*, we first predicted their subcellular localization (Table S3). We found that the 144 *F-box* genes were unevenly distributed across different cellular compartments, including the nucleus, cytoplasm, extracellular space, plasma membrane, mitochondria, Golgi, peroxisomes, and vacuoles. Specifically, the proportion of *F-box* genes distributed in these structures is 29:16:8:19:22:2:3:1 (Figure 6B). Moreover, we found that some subfamily genes were localized in various cellular structures. For instance, the *FBL* subfamily was distributed in cytoplasmic, nuclear, and peroxisomal cellular structures, while the *FBX* subfamily was distributed across all eight cellular structures to some extent (Figure 6A).

To further understand the biological functions of the *F-box* gene-encoded proteins in *L. chinense*, we conducted gene ontology analysis to predict the function of homologous genes based on studies of *F-box* genes in *A. thaliana*. We found that the majority of *L. chinense* F-BOX was identified as being involved in protein-binding molecular functions (Table S4), which was consistent with previous studies. Additionally, we found that 12 genes had multiple GO numbers, indicating that these *F-box* genes were involved in specific biological processes (Table 1).

We also conducted KEGG pathway enrichment analysis, which revealed that the majority of *L. chinense F-box* genes (35) were involved in the biological process of ubiquitination, consistent with their function as components of the SCF complex. Additionally, 13 genes were predicted to be involved in starch and sucrose metabolism, 12 in ascorbate and aldarate metabolism pathways, 9 in signaling protein pathways, and 7 in the transcription machinery pathway, among others (Figure 6C).

**Figure 7:**
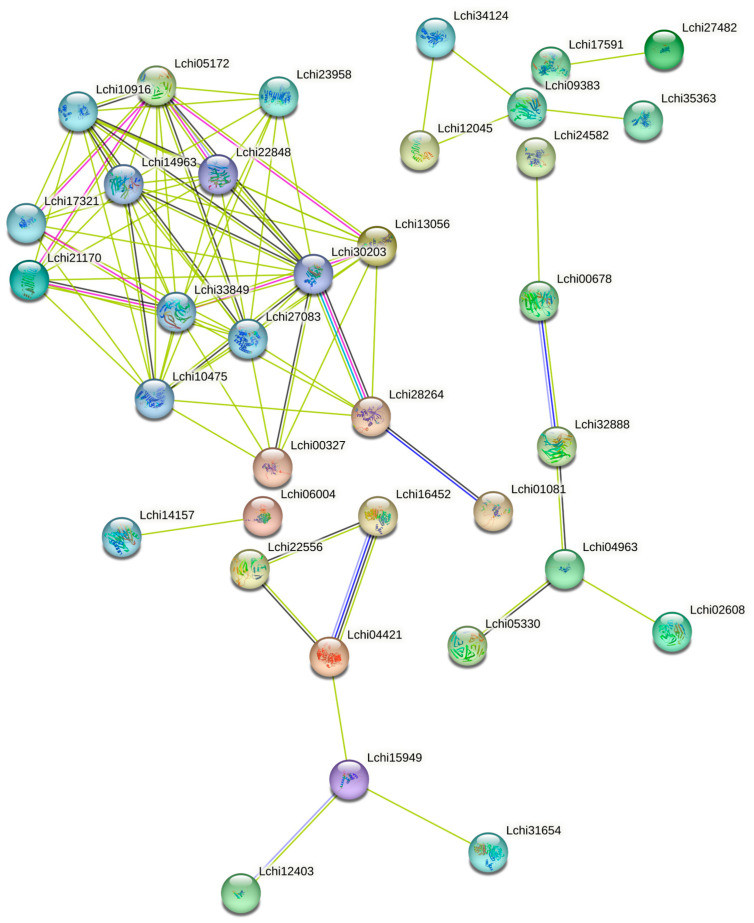
Predicted protein–protein interaction network for the *F-box* gene in *L. chinense*. Different line colors represent different types of protein–protein interactions. Colored nodes: query proteins and the first shell of interactors; filled nodes: some 3D structure is known or predicted.

The interaction between 144 F-BOXs was predicted by the STRING online website (Figure 7). The results showed that 35 F-BOX genes may interact, and 15 genes form the key nodes of the interaction network. They are *Lchi10916*, *Lchi27083*, *Lchi10475*, *Lchi28264*, *Lchi01081* of the *FBXL* subfamily; *FBX* subfamily *Lchi05172*, *Lchi17321*, *Lchi14963*, *Lchi00327*; *Lchi21170*, *Lchi23958*, *Lchi13056* of the *FBD* subfamily; *Lchi22848*, *Lchi33849* of the *FBA* subfamily; and *Lchi30203* of the *FBL* subfamily.

### 2.4. F-Box Gene Expression Levels across Different Tissue Types in Liriodendron Hybrids

**Figure 8:**
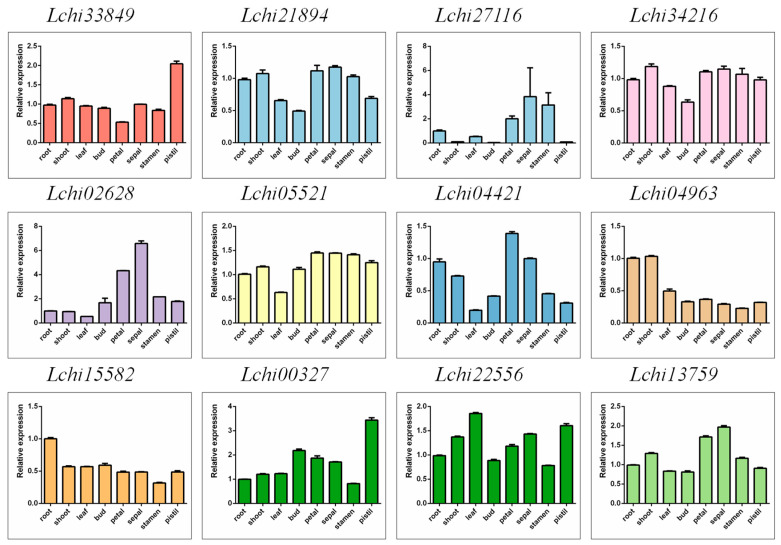
Expression patterns of *L. chinense F-box* family genes in different tissues were analyzed by qRT-PCR.

To investigate the expression patterns of the *F-box* gene family, we obtained transcriptome data for different tissues of *Liriodendron* hybrids from the NCBI website. We extracted the *F-box* family genes and used them to generate a heatmap of their expression patterns (Figure S1, Table S5). Relatively speaking, most genes tend to be highly expressed in buds and pistils and lowly expressed in leaves and petals. A small number of genes are highly expressed in stamens, most of which belong to the FBA subfamily, indicating that the FBA subfamily genes may be related to some functions of stamens (Figure S1). The cluster analysis of gene expression showed that the tissue-specific expression patterns of *F-box* genes were concentrated in 14, 9, and 7 patterns, and the number of genes contained in the three patterns was 26, 22, and 11, respectively (Figure S2). To verify the reliability of the transcriptome data, we selected 1–2 genes (selection criteria: 1. the expression level of the gene cannot be too low to prevent the gene from being detected by qRT-PCR; 2. the designed primers were amplified by semi-quantitative PCR, and the product had a target band) from each subfamily for qRT-PCR analysis. We found that different subfamily genes showed different expression patterns in different tissues. For instance, *Lchi33849* of the *FBA* subfamily had relatively high expression in stamens and relatively low expression in petals. We also observed that *Lchi34216* of the *FBDUF* subfamily had relatively low expression in buds, *Lchi02628* of the *FBK* subfamily had high expression in sepals and relatively low expression in leaves, and *Lchi04963* of the *FBT* subfamily had high expression in roots and stems but relatively low expression in other tissues, among others. Additionally, different genes within the same subfamily showed different expression patterns in different tissues, such as *Lchi21894* and *Lchi27116* of the *FBD* subfamily and *Lchi00327* and *Lchi22556* of the *FBX* subfamily (Figure 8). In summary, we speculate that the specific expression of *F-box* genes in different tissues means that these genes may be related to the developmental regulation of specific tissues.

### 2.5. F-Box Genes Are Universally Expressed at Different Stages of Somatic Embryogenesis in Liriodendron Hybrids

**Figure 9:**
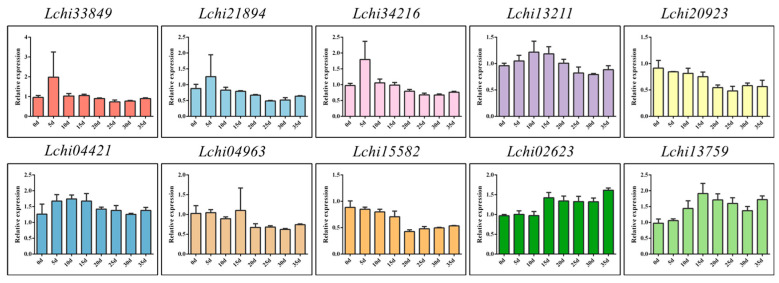
Expression patterns of *L. chinense F-box* family genes during somatic embryogenesis of *liriodendron* hybrids were analyzed by qRT-PCR.

To further understand the role of *F-box* family genes in affecting plant development, we obtained expression data for the 144 *F-box* genes from the transcriptome data of somatic embryogenesis in *Liriodendron* hybrids (Figure S3, Table S6). During the whole process of somatic embryogenesis, the expression of the *F-box* gene is differentially regulated. Some are up-regulated in the early stages, while others are up-regulated in both the early and late stages. In addition, a small number of genes are highly expressed in the middle stage (Figure S3). Cluster analysis of these expression patterns showed that with the development of somatic embryos, these *F-box* genes were concentrated in five expression patterns, and 28 genes were clustered into two expression patterns, which showed that the expression decreased first, then increased, and finally decreased. Furthermore, 29 genes were clustered into 19 expression patterns, which showed that their expression levels continued to increase. Eighteen genes were clustered into one type of expression pattern, and their expression levels decreased first and then increased. The 16 genes were clustered into zero expression patterns, and their expression levels showed a continuous decrease. The 16 genes were clustered into nine expression patterns, and their expression levels were similar to those of the two expression patterns (Figure S4). We conducted qRT-PCR verification and found that, during the somatic embryogenesis of *Liriodendron* hybrids, *Lchi20923* and *Lchi15582* showed a decreasing trend, and the expression of *Lchi04963* decreased first, then increased, and then increased. In addition, other genes showed a trend of increasing first, then decreasing, and then increasing (Figure 9). In summary, the change in *F-box* gene expression during somatic embryogenesis indicates that it may be involved in some biological processes during somatic embryogenesis and thus plays a regulatory role in somatic embryo development.

### 2.6. F-Box Responds Strongly to Cold, Heat, and Drought Stress Treatments in Liriodendron Hybrids

**Figure 10:**
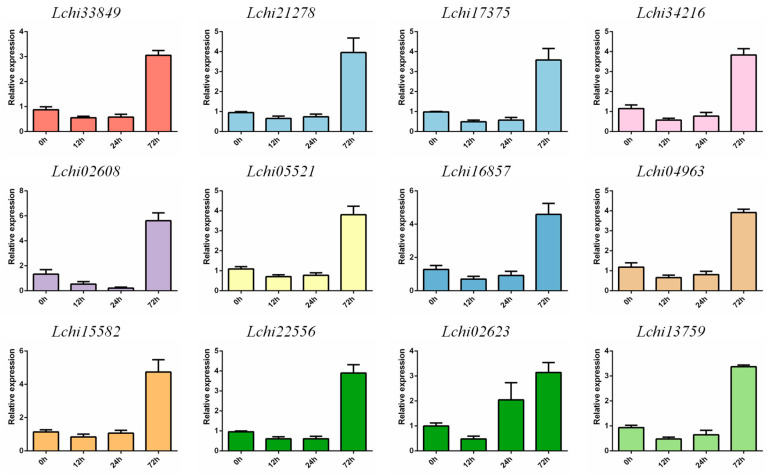
The expression pattern of the *F-box* gene under drought stress was analyzed by qRT-PCR.

Numerous studies have shown that *F-box* family genes play significant roles in stress responses. Therefore, we extracted transcription data for these 144 genes under drought, high temperature, and cold stress (Figures S5, S7 and S9; Table S7). In cold stress, the peak expression of some genes appeared at 0 h, while the peak expression of other genes appeared at 3 day (Figure S5). Its expression patterns are basically clustered between 0 and 19. The expression pattern 0 contained 45 genes, and the expression level continued to increase. Expression pattern 19 included 11 genes, and the expression level showed a continuous decrease (Figure S6). In heat stress, the peak of *F-box* gene expression appeared at 0 day, 1 day, and 3 day (Figure S7). Its expression patterns are clustered into 1 and 19 patterns. The expression pattern 1 contained 25 genes, and the expression level decreased first and then increased. The expression pattern 19 contains 19 genes, and the expression level is continuously reduced (Figure S8). Under drought stress, the peak of *F-box* gene expression appeared at multiple time points (Figure S9). Its expression patterns are clustered into zero and six patterns. The expression pattern 0 contained 17 genes, and the expression level showed a continuous decrease. The expression level 6 contained 12 genes, and the expression level showed a repeated fluctuation of decreasing first and then increasing (Figure S10). Then we conducted qRT-PCR verification of gene expression under drought stress. Interestingly, we found that during the process of drought stress, the gene expression levels of almost all subfamilies first decreased and then increased, reaching a peak expression level at 72 h of drought treatment (Figure 10). In summary, the differential expression of *F-box* genes in these stress responses indicates that they may be regulated by certain factors in the process of stress response, thus responding to these abiotic stresses.

## 3. Discussion

The *F-box* gene family, which is involved in many biological pathways, is one of the largest gene families in eukaryotes and plays a critical role in the complete life cycle of plants, making its study essential for understanding plant growth and development.

**Figure 11:**
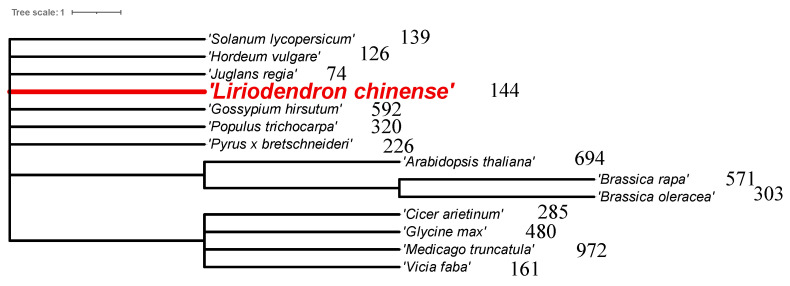
The number of *F-box* genes in different species—the number after the Latin name of a species—indicates the number of *F-box* genes in that species.

The *F-box* gene family has been extensively analyzed and studied in various species, with the number of its members varying widely across species (Figure 11). This variability may be attributed to the different identification thresholds and criteria adopted by different researchers [12]. For instance, the number of *F-box* gene family members in rice was reported to be 680, 858, 764, while in *A. thaliana* it was 660,698, and in aspen, it was 320, 337, 387 [8,35,36]. Furthermore, differences in the number of *F-box* gene family members may be a result of gene duplication during species evolution, which could lead to both gene loss and expansion. Gene replication is considered an essential contributor to gene family expansion and functional diversity during evolution, and it may occur through chromosome segmental replication or tandem replication [37]. In chickpeas, for instance, repeats made up 43.7% of the 192 *F-box* genes, of which 38 (13.3%) resulted from segmentary repeats and 62 (21.8%) arose from tandem repeats. These findings suggest that tandem repeats, rather than segmentary repeats, played a more significant role in the expansion of the *F-box* gene family in chickpeas [11]. In *L. chinense*, there are also eight pairs of tandem repeats in the *F-box* gene family. Among the eight gene pairs, five pairs belong to the *FBD* subfamily, while previous studies have shown that the *F-box* gene subfamily shows bias in its amplified repeat pattern, and most genes involved in tandem and segmental duplications belong to the *FBD*, *FBX*, and *FBL* subfamilies [11].

The functional diversity of *F-box* genes is closely linked to the conserved C-terminal domain of the *F-box* genes. In addition to the conserved F-BOX domain, the C-terminal of the *F-box* gene usually contains a Kelch repeat, a leucine-rich repeat, the FBD domain, the FBA domain, Tubby, PP2, and other conserved domains. These domains have distinct biological functions. For example, the Kelch repeat domain is involved in circadian clock regulation, recognizes substrates, and mediates their ubiquitination [38]. LRR is associated with plant resistance, and in *A. thaliana*, LRR is able to recognize flagellin (flg22) in the defense response, thus initiating plant resistance [39,40]. The WD40 domain promotes flavonoid biosynthesis in protein-interacting plants [41,42,43]. The FBA domain is involved in carbohydrate metabolism, signal transduction [44,45,46,47], and so on. Here, we divided *F-box* family genes into 10 subfamilies according to these conserved domains. However, in *L. chinense*, we did not find the WD40 domain, which we speculated may be due to the ancient evolutionary status of *L. chinense*, and the WD40 domain may have evolved later.

To delve further into the gene function of the *F-box* gene family in *L. chinense*, we predicted the subcellular locations of these genes and found that most are located in the nucleus, followed by the mitochondria, plasma membrane, and cytoplasm. Interestingly, different subfamilies show a certain bias in their predicted subcellular locations; for instance, the *FBL* subfamily is not predicted to be located in other cell structures aside from the nucleus, cytoplasm, and peroxisomic structures. The annotation structure of the gene body showed that most of the *F-box* family genes were annotated to the protein-binding (GO:0005515) module, indicating that *F-box* genes may play a key role in gene transcription regulation of pear, which is consistent with previous studies [9,12]. KEGG enrichment results showed that most *F-box* genes (35) were involved in the biological process of ubiquitination, which is related to the fact that F-BOX is one of the most typical ubiquitin ligases in the SCF complex [2,3].

Gene expression analysis is an insightful tool for studying the functionality of genes, and RNA-seq is a powerful technique for studying the transcription patterns of specific genes through high-throughput sequencing. To explore the tissue expression specificity of *F-box* genes, we employed published RNA-seq data and studied *F-box* gene expression profiles under cold, heat, and drought stress in order to understand the *F-box* gene responses to these stresses. Moreover, to investigate the role of *F-box* genes in plant growth and development, the transcriptome data of *Liriodendron* hybrid embryogenesis was analyzed. The q-RCR results revealed that during somatic embryogenesis, most *F-box* genes exhibited a pattern of initial increase, then decrease, and finally increase again. This indicates that these genes may be involved in the initiation of embryonic development and postembryonic growth. Furthermore, during drought stress treatment, the expression levels of these *F-box* genes decreased initially and then increased, reaching their highest level after 72 h of stress treatment. This suggests that *F-box* genes may be upregulated during this period to resist the effects of drought stress.

## 4. Materials and Methods

### 4.1. Database Search for F-Box Proteins in L. chinense

To identify all the protein sequences in the *L. chinense* genome containing F-BOX and F-BOX-LIKE domains, we downloaded the *L. chinense* genomic dataset from the *L. chinense* genomic assembly data (https://db.cngb.org/search/project/CNP0000815/) (accessed on 23 May 2020) [29]. We then obtained the Hidden Markov Model (HMM) files of F-BOX (PF00646) and F-BOX-LIKE (PF12937) from the Pfam database (http://pfam.xfam.org) (accessed on 5 December 2020) [48]. HMMER v3.2.1 (https://www.ebi.ac.uk/Tools/hmmer/) (accessed on 5 December 2020) [49] with an E-value cutoff of 1.0 was used as a query tool to search for all protein sequences containing F-BOX and F-BOX-LIKE in the *L. chinense* genome. Additionally, we used CDD (Conservative Domain Database) (https://www.ncbi.nlm.nih.gov/Structure/cdd/wrpsb.cgi) (accessed on 5 December 2020) [50], SMART (http://smart.embl-heidelberg.de/) (accessed on 5 December 2020) [51], and Pfam to identify the conserved domain of the F-BOX protein with an E-value cutoff value of 1.0.

### 4.2. Sequence Analysis

After obtaining the F-BOX sequences in *L. chinense*, we performed alignment of all F-BOX motifs using ClustalW in MEGA-X (v10.1.8) [52]. Sequential logos were generated using the online program Weblogo (http://weblogo.berkeley.edu/logo.cgi) (accessed on 16 December 2020) [53]. Furthermore, we submitted all the C-terminal F-BOX sequences of *L. chinense* that had an unknown domain to the MEME (Multiple Expectation Maximization for Motif Elicitation) website (https://meme-suite.org/meme/) (accessed on 15 December 2020) [54] to identify the unknown conserved domain. We also determined the basic properties, including the length, molecular weight (MW), and isoelectric point (PI), of the F-BOX family using ExPasy (https://web.expasy.org/compute_pi/) (accessed on 13 March 2021) [55].

### 4.3. Phylogenetic Analysis and Gene Structure Analysis

We used the ClustalW program of MEGA-X software (v10.1.8) [52] to perform multiple sequence alignments of 144 F-BOX in *L. chinense* and constructed a phylogenetic tree using the maximum likelihood method. The estimation was carried out using the Jones-Thornton-Taylor (JTT) model algorithm, and bootstrap analysis was performed with a repeated value of 1000. We visualized the phylogenetic tree using the online program ITOL (https://itol.embl.de/) (accessed on 15 December 2020) [56].

We used the online software MEME (http://meme-suite.org/) (accessed on 15 December 2020) to analyze the motif types and sequences of the *F-box* gene family members in *L. chinense* and obtained the motif characteristics of the gene family. Using the genomic annotation information, we visualized the gene structure and motif of the *F-box* gene using TBtools software [57].

### 4.4. Chromosomal Locations, Gene Duplication Analysis, and Synteny Analysis

We obtained the physical location of all *F-box* genes from the *L. chinense* genome annotation files and used TBtools [57] to analyze and visualize the tandem repeat and synteny of all *F-box* genes.

### 4.5. Subcellular Localization, GO Enrichment Analysis, and KEGG Enrichment Analysis

We used the online software ProtComp 9.0 (http://linux1.softberry.com/berry.phtml?topic=protcompan&group=programs&subgroup=proloc) (accessed on 18 January 2021) to analyze the amino acid sequences of each member of the candidate *F-box* gene family and predict their subcellular localization.

We obtained the Gene Ontology (GO) annotation for *L. chinense* F-BOX-encoding genes from the *L. chinense* genome project [29]. We analyzed the top three GO categories: molecular function (MF), biological process (BP), and cellular component (CP). Additionally, we predicted the functional annotations of *F-box* genes involved in any biological process (BP) based on putative homologues from *A. thaliana*.

We obtained the KEGG (Kyoto Encyclopedia of Genes and Genomes) enrichment analysis for *L. chinense* F-BOX-encoding genes from the *L. chinense* genome project [29].

### 4.6. RNA-Seq Analysis of F-Box Gene Expression Levels in Different Organs and Multiple Stresses

To investigate the expression patterns of the *F-box* gene family, we downloaded transcript data from NCBI for different organs of *L. chinense*, as well as data on responses to high temperature (0 h, 1 h, 3 h, 6 h, 12 h) and drought (0 h, 1 h, 3 h, 6 h, 12 h, 24 h, and 72 h) stresses. We also obtained transcript data for low temperature (4 °C (0 h, 12 h, 24 h, and 48 h)) and *Liriodendron* hybrid somatic embryogenesis (ES2: 2 days after screening; ES3: ABA 1 day of treatment; ES4: ABA treatment for 3 days; ES5: globular embryo; ES6: heart-shaped embryo; ES7: torpedo embryo; ES8: immature cotyledon embryo; ES9: mature cotyledon embryo; PL: plantlet) (unpublished data). All mRNA abundance values were measured by transcripts per million (TPM) based on the *L. chinense* genomic database.

### 4.7. Plant Materials, Treatments, and Collections; RNA Extraction and Quantitative Real-Time PCR Analysis

For our experimental validation of the expression patterns of the *F-box* gene family in *L. chinense*, we collected different tissues, including roots, stems, leaves, buds, petals, sepals, stamens, and pistils. We also exposed plants to 15% PEG drought treatment for 0 h, 12 h, 24 h, and 72 h and monitored somatic embryogenesis for 0 day, 5 day, 10 day, 15 day, 20 day, 25 day, 30 day, and 35 day. Total RNA from these samples was extracted using the Total RNA Isolater Total RNA Extraction Reagent. We used the Vazyme HiScript III 1st Strand cDNA Synthesis Kit (+gDNA wiper) to convert the RNA to cDNA, which was then diluted to 160 μL. We performed fluorescent quantitative PCR on a Roche Applied Science LightCycler 480 using Vazyme’s AceQ qPCR SYBR Green Master Mix (without ROX) reagent, and each sample was repeated three times. We used ACT97 as the internal reference gene, and all primer sequences are shown in Table S8.

## 5. Conclusions

In this study, the *F-box* genes in the genome of *L. chinense* were comprehensively analyzed, and 144 *F-box* genes were identified. Bioinformatics and qRT-PCR were used to analyze the gene structure, phylogeny, chromosome localization, gene replication, subcellular localization, GO annotation, and KEGG enrichment analysis of *L. chinense F-box* family genes. The differential expression of *F-box* family genes in different tissues, somatic embryogenesis, and different stress responses of *L. chinense* indicated that they played an important role in the growth and development of *L. chinense* and stress responses. This study provides comprehensive information on the *F-box* genes of *L. chinense*, which will help to further study the function of different *F-box* genes.

## Figures and Tables

**Table 1:** The 12 *F-box* genes involved in biological processes (BPs) based on the known functions of orthologous *Arabidopsis* genes.

Gene_Id	Go Number	*Arabidopsis* Ortholog Genes	Putative Function of *Arabidopsis* Orthologs
Lchi01743	3	AT5G39450	biological process
Lchi01886	4	AT1G21890	enables transmembrane transporter activity
Lchi03271	2	AT5G57360	flower development and protein ubiquitination
Lchi14963	6	AT5G43190	ubiquitin-dependent protein catabolic process
Lchi15076	6	AT5G43190	ubiquitin-dependent protein catabolic process
Lchi17591	3	AT3G54460	enables ATP-dependent chromatin remodeling activity
Lchi23560	2	AT2G17020	ubiquitin-dependent protein catabolic process
Lchi26033	2	AT1G12870	regulation of transcription
Lchi31059	2	AT1G68050	positive regulation of flower development
Lchi31193	4	AT4G08850	hormone-mediated signaling pathway
Lchi33849	2	AT1G13200	regulation of transcription
Lchi34026	2	AT1G23770	biological process

## Data Availability

Transcriptome data on somatic embryogenesis and tissues have not yet been published. The abiotic stress transcriptome data of *Liriodendron* hybrids are annotated with accession number PRJNA679101 and can be downloaded through NCBI (https://www.ncbi.nlm.nih.gov/bioproject/PRJNA679101/, accessed on 7 August 2022). The complete genome, transcript/protein sequences, and genome feature files of Lchi were downloaded from https://www.ncbi.nlm.nih.gov/assembly/GCA_003013855.2, accessed on 7 August 2022.

## Supplementary Materials

The following supporting information can be downloaded at: https://www.mdpi.com/article/10.3390/plants13020171/s1. Figure S1: Transcriptome data heat map of different tissues of *Liriodendron* hybrids; Figure S2: Gene number cluster analysis of RNA-seq expression trend in different tissues of *F-box* genes. Note: The top-left value indicates the ID of the trend, and the bottom-left value indicates the number of genes in the trend. Colored trend block: a significant enrichment trend. The different colors are set by the software to distinguish different trends, and the colors have no special meaning. Trend block without color: non-significant enrichment trends. Figure S3: Transcriptome data heat map during somatic embryogenesis of *Liriodendron* hybrids; Figure S4: The gene number cluster analysis of RNA-seq expression trend in somatic embryogenesis of *F-box* genes. Figure S5: Transcriptome data heat map of *Liriodendron* hybrids under drought stress; Figure S6: The gene number cluster analysis of RNA-seq expression trend in drought stress of *F-box* genes. Figure S7: Transcriptome data heat map of *Liriodendron* hybrids under cold stress; Figure S8: The gene number cluster analysis of RNA-seq expression trend in cold stress of *F-box* genes. Figure S9: Transcriptome data heat map of *Liriodendron* hybrids under heat stress. Figure S10: The gene number cluster analysis of the RNA-seq expression trend in heat stress of *F-box* genes. Table S1: The basic characteristics of the *F-box* gene in *L. chinense*; Table S2: The number of introns, exons, and UTR of the *F-box* gene in *L. chinense*; Table S3: Subcellular localization prediction of the *F-box* gene in *L. chinense*; Table S4: GO function annotation of the *F-box* gene in *L. chinense*. Table S5: Transcriptome data of the *F-box* gene expression in different tissues of *Liriodendron* hybrids; Table S6: Transcriptome data of the *F-box* gene in the somatic embryogenesis of *Liriodendron* hybrids; Table S7: Transcriptome data of *F-box* genes in *Liriodendron* hybrids under drought, high temperature, and cold stress; Table S8: Primer sequences for qRT-PCR.
