# Supplementary figures and images for: The Identification and Expression Analysis of the Liriodendron chinense F-Box Gene Family
Source: Plants (Basel). 2024 Jan 8;13(2):171. doi: 10.3390/plants13020171 (PMC10819036; doi:10.3390/plants13020171)

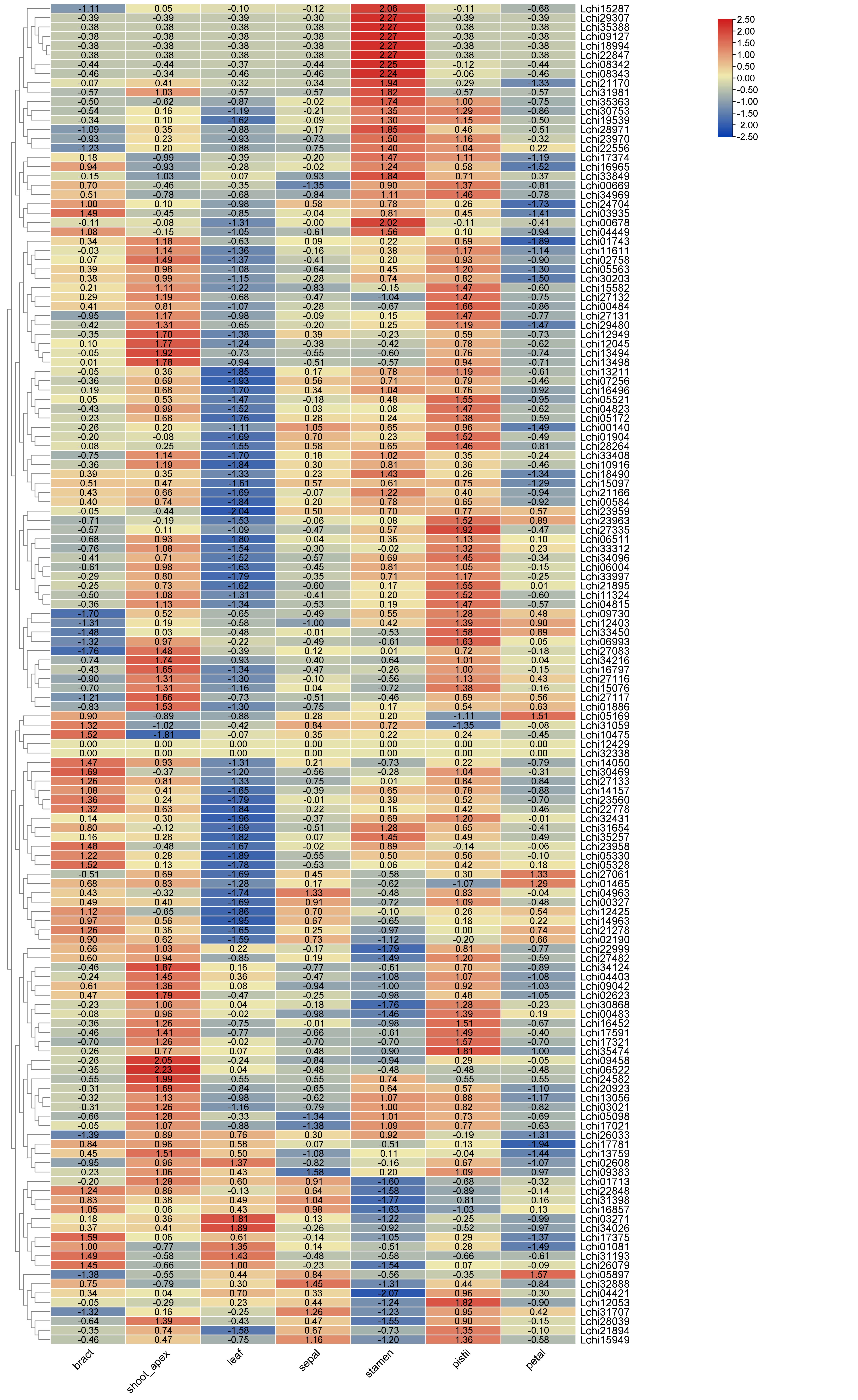

Supplement: Supplementary file 1 [file plants-13-00171-s001.zip › Figures S1-S10/Figure S1.jpg]

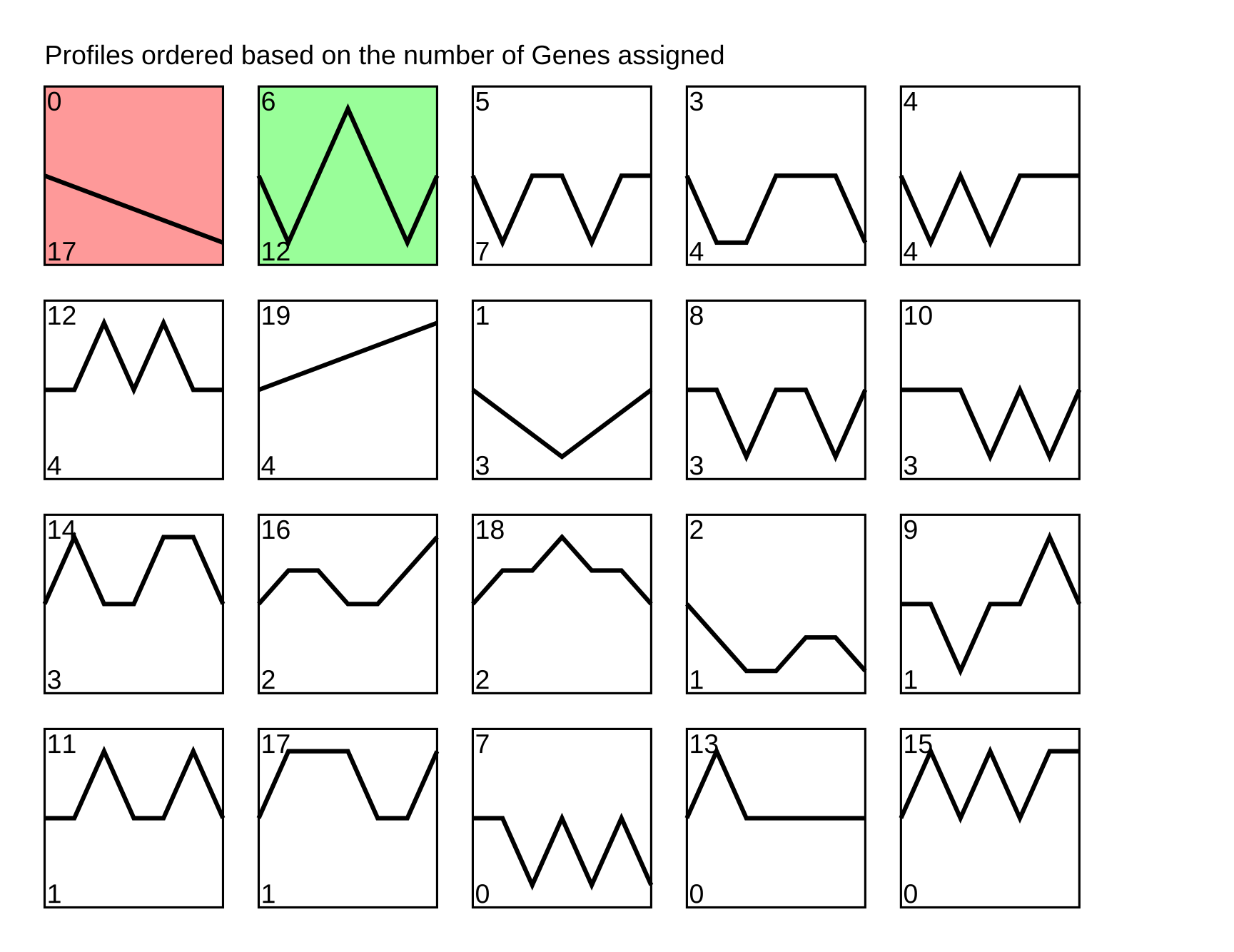

Supplement: Supplementary file 1 [file plants-13-00171-s001.zip › Figures S1-S10/Figure S10.png]

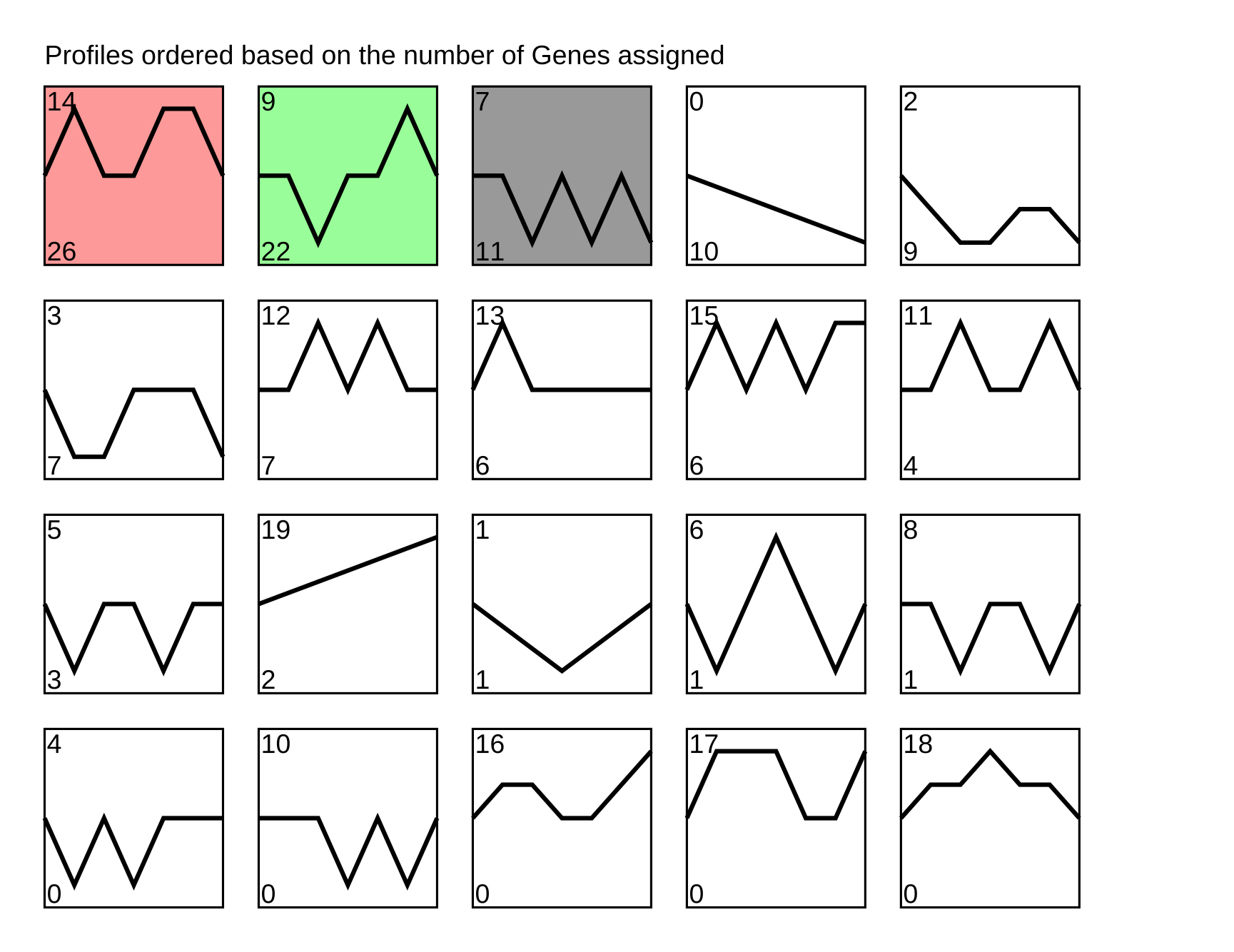

Supplement: Supplementary file 1 [file plants-13-00171-s001.zip › Figures S1-S10/Figure S2.png]

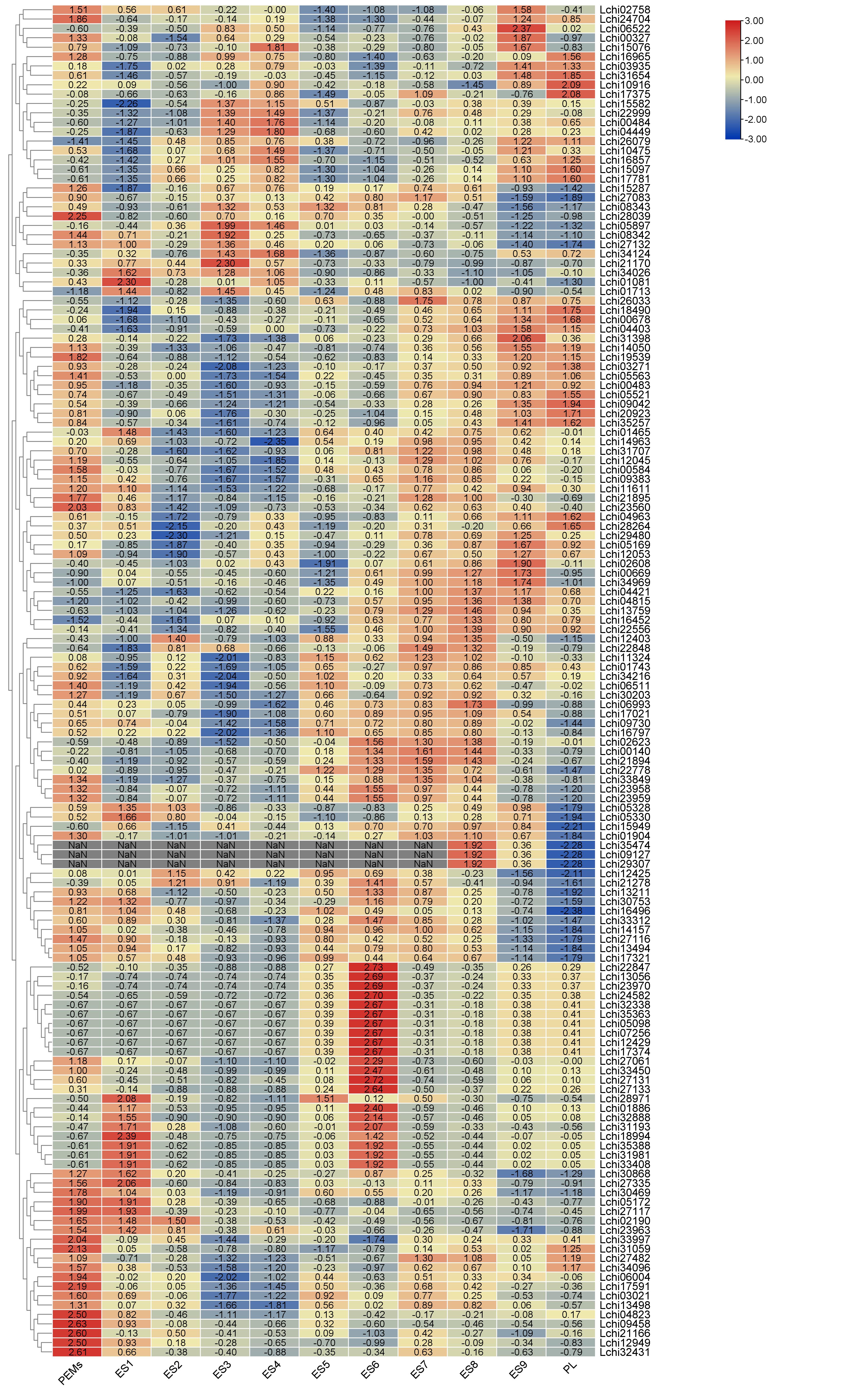

Supplement: Supplementary file 1 [file plants-13-00171-s001.zip › Figures S1-S10/Figure S3.jpg]

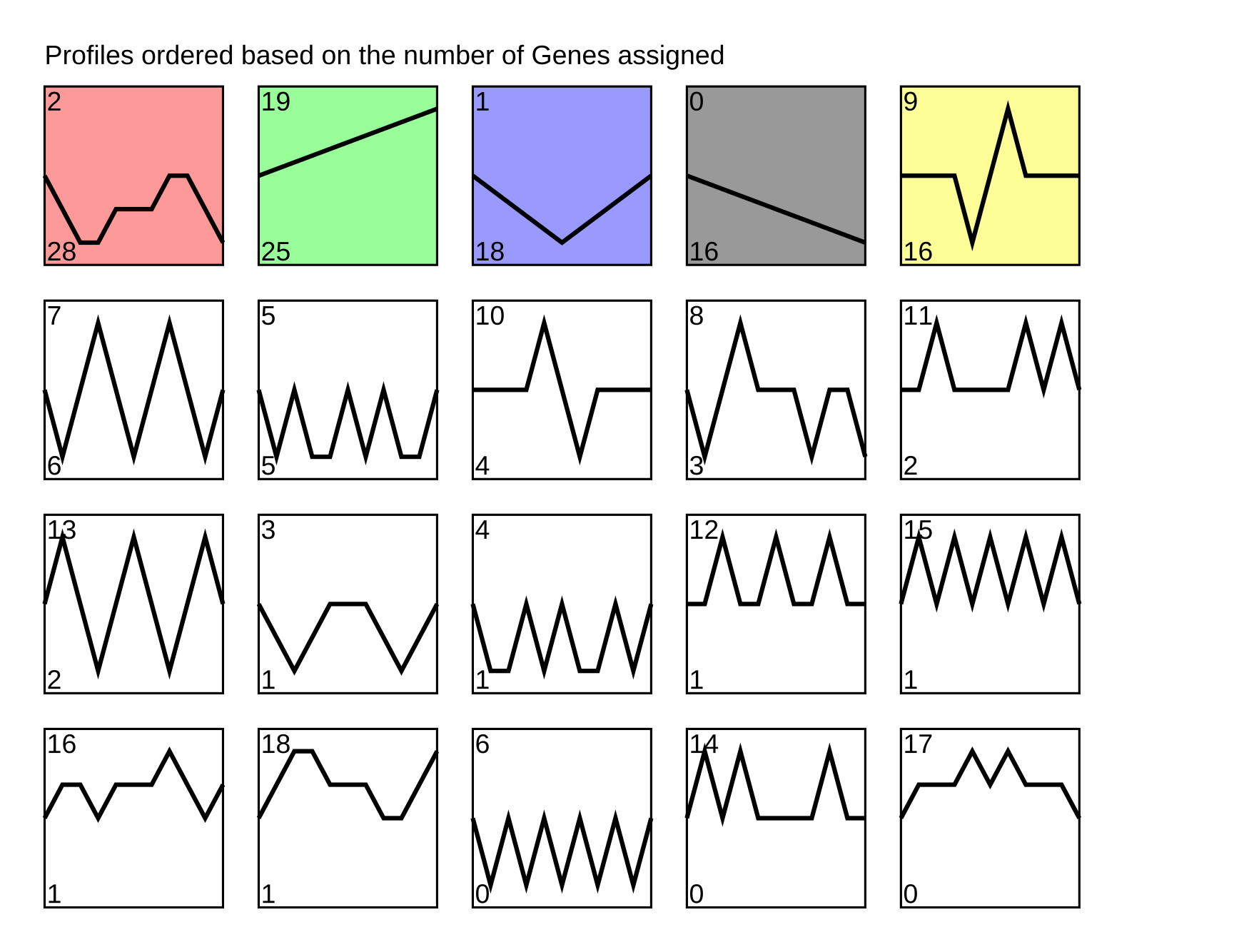

Supplement: Supplementary file 1 [file plants-13-00171-s001.zip › Figures S1-S10/Figure S4.png]

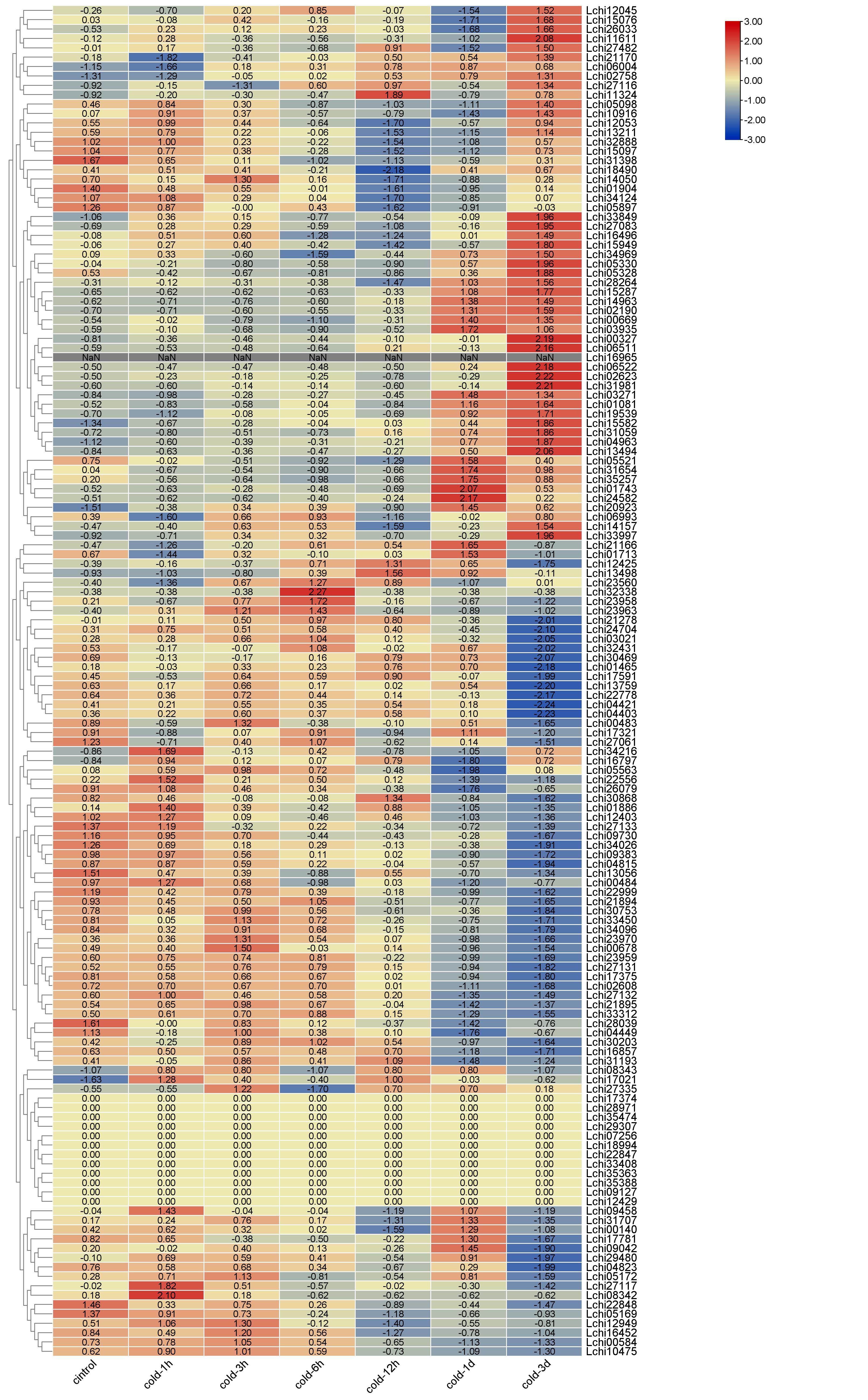

Supplement: Supplementary file 1 [file plants-13-00171-s001.zip › Figures S1-S10/Figure S5.jpg]

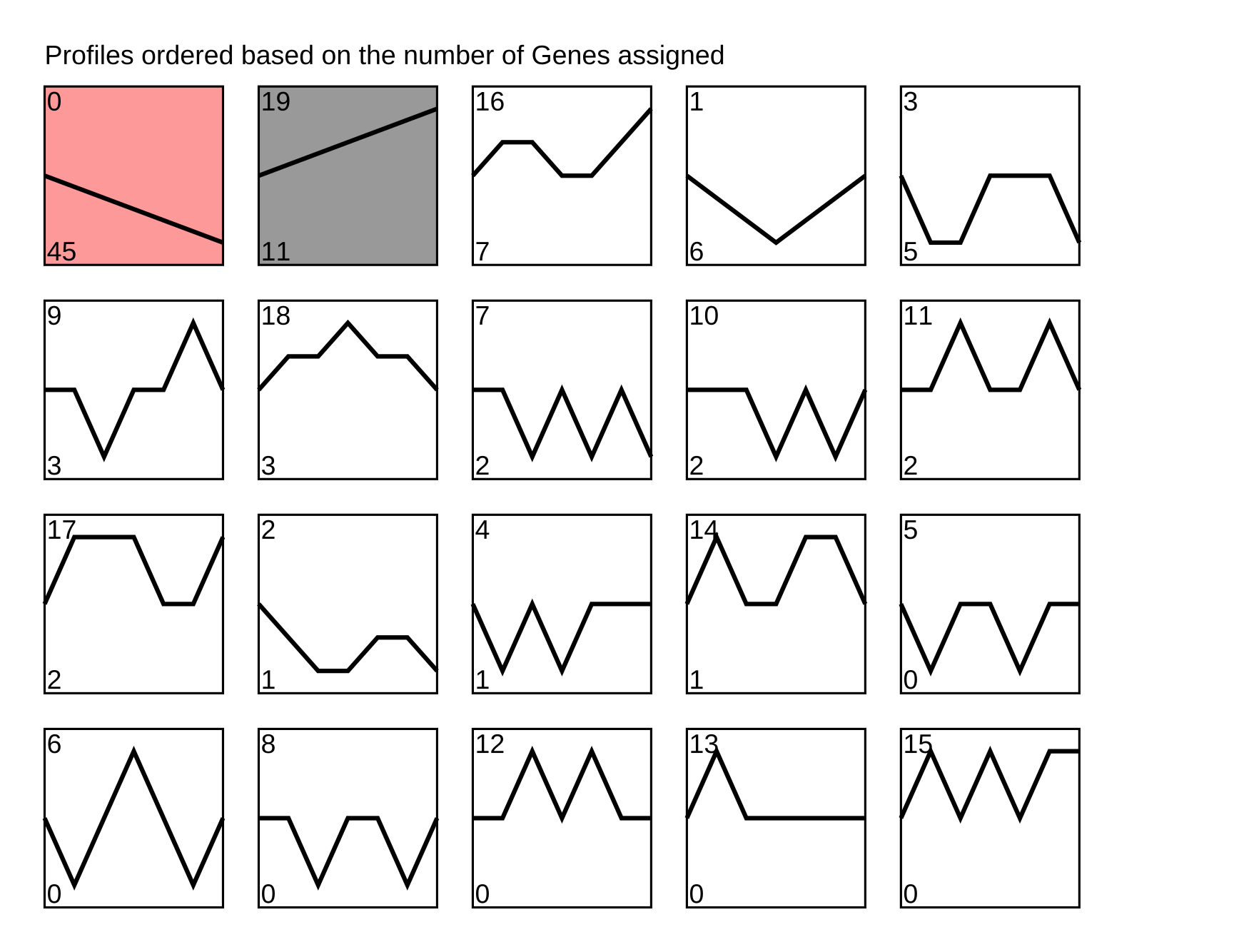

Supplement: Supplementary file 1 [file plants-13-00171-s001.zip › Figures S1-S10/Figure S6.png]

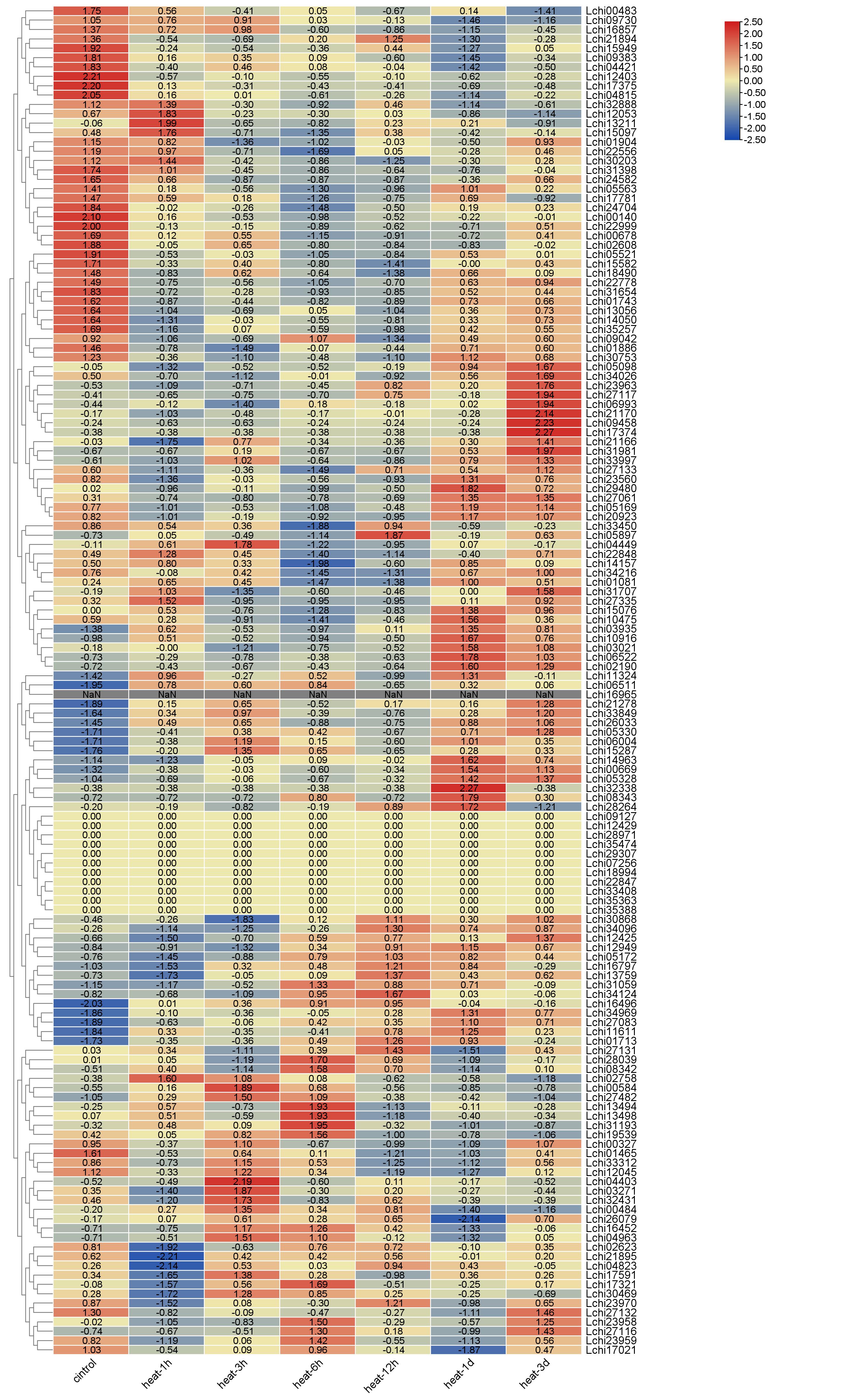

Supplement: Supplementary file 1 [file plants-13-00171-s001.zip › Figures S1-S10/Figure S7.jpg]

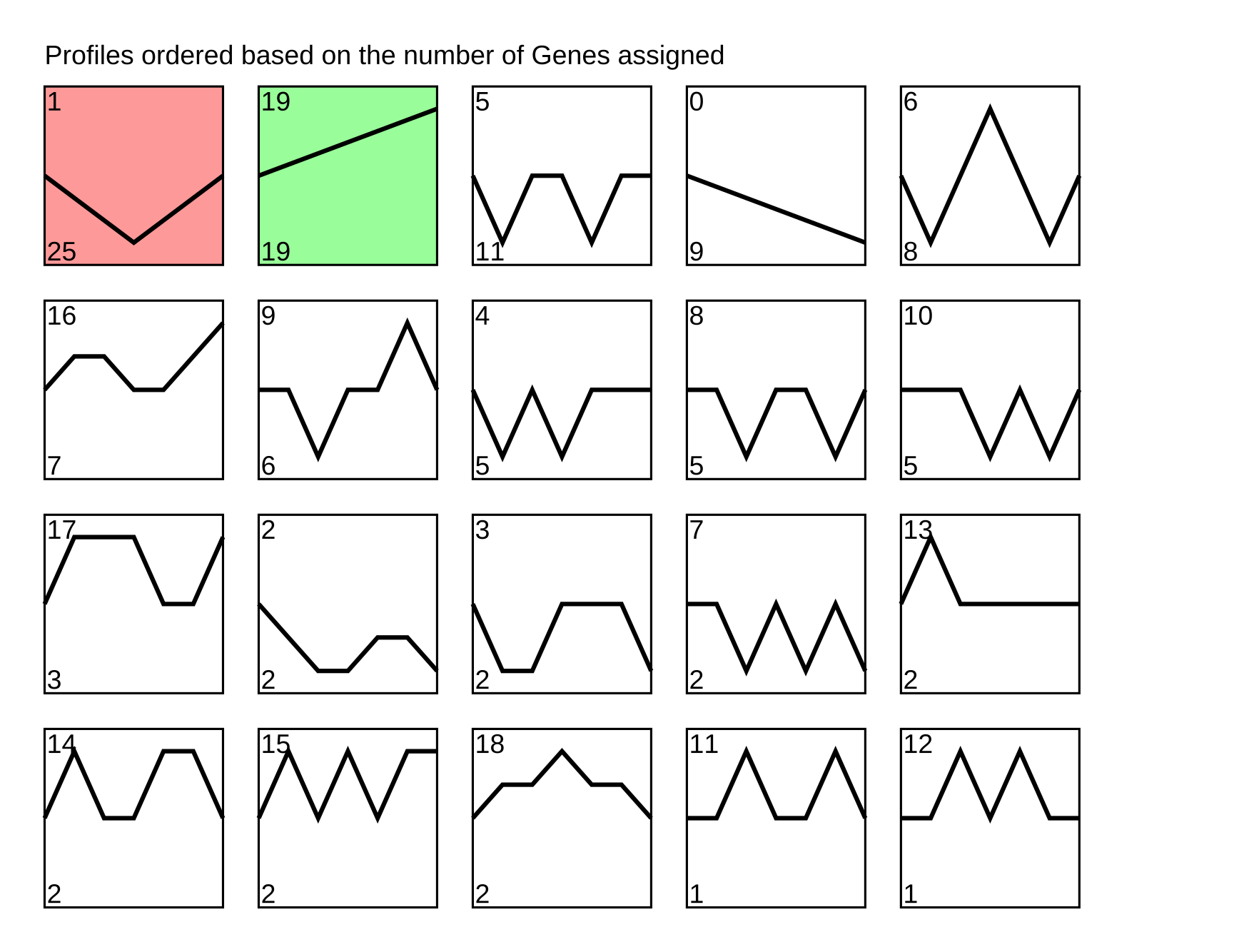

Supplement: Supplementary file 1 [file plants-13-00171-s001.zip › Figures S1-S10/Figure S8.png]

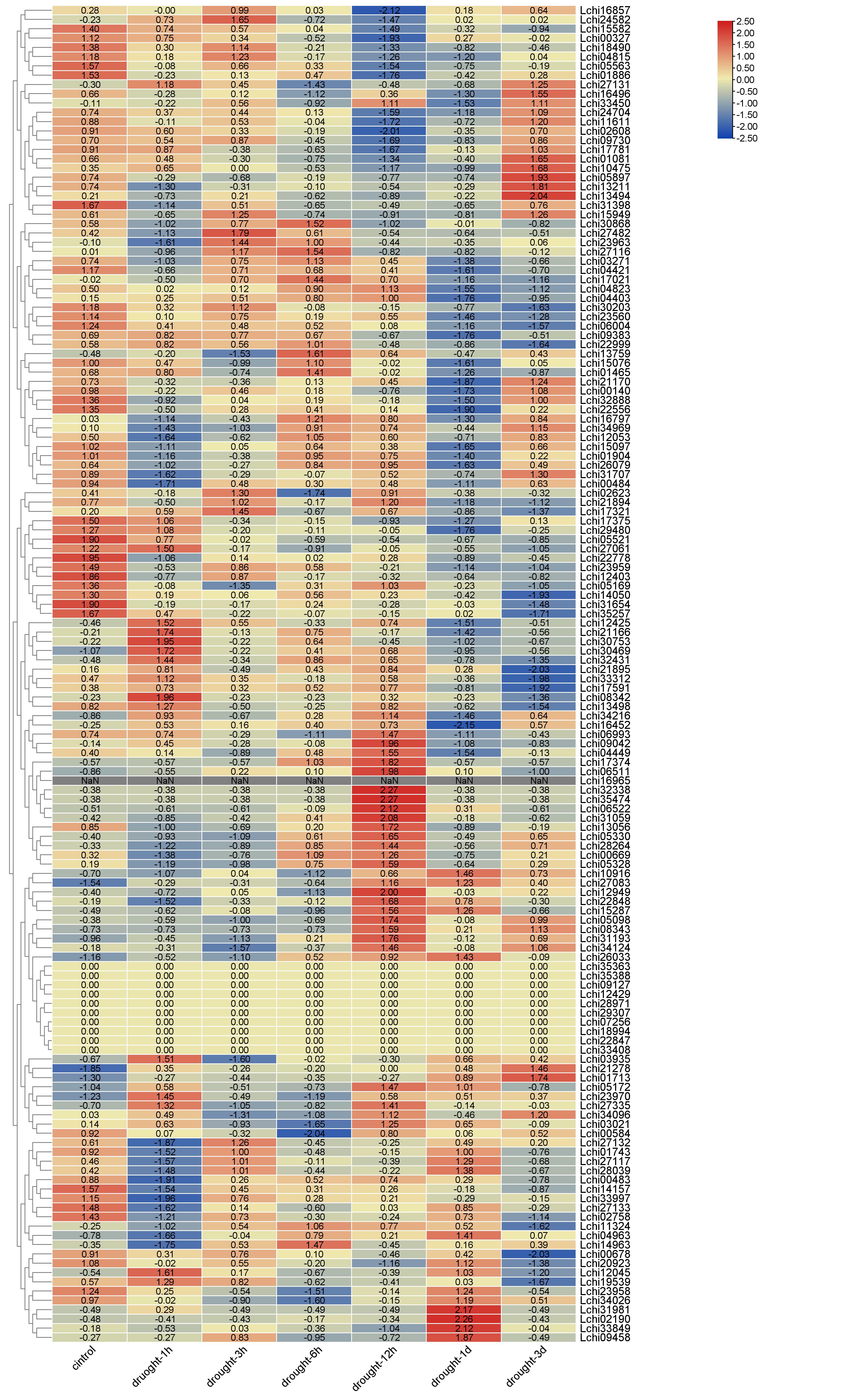

Supplement: Supplementary file 1 [file plants-13-00171-s001.zip › Figures S1-S10/Figure S9.jpg]
